# A simulation study to compare different estimation approaches for network meta-analysis and corresponding methods to evaluate the consistency assumption

**DOI:** 10.1186/s12874-020-0917-3

**Published:** 2020-02-24

**Authors:** Corinna Kiefer, Sibylle Sturtz, Ralf Bender

**Affiliations:** 1 Institute for Quality and Efficiency in Health Care (IQWiG), Im Mediapark 8, Cologne, D-50670 Germany; 2 Faculty of Medicine, University of Cologne, Joseph-Stelzmann-Str. 20, Cologne, D-50931 Germany

**Keywords:** Consistency assumption, Indirect comparison, Mixed treatment comparison, Multiple treatments meta-analysis, Network meta-analysis, Simulation study

## Abstract

**Background:**

Network meta-analysis (NMA) is becoming increasingly popular in systematic reviews and health technology assessments. However, there is still ambiguity concerning the properties of the estimation approaches as well as for the methods to evaluate the consistency assumption.

**Methods:**

We conducted a simulation study for networks with up to 5 interventions. We investigated the properties of different methods and give recommendations for practical application. We evaluated the performance of 3 different models for complex networks as well as corresponding global methods to evaluate the consistency assumption. The models are the frequentist graph-theoretical approach netmeta, the Bayesian mixed treatment comparisons (MTC) consistency model, and the MTC consistency model with stepwise removal of studies contributing to inconsistency identified in a leverage plot.

**Results:**

We found that with a high degree of inconsistency none of the evaluated effect estimators produced reliable results, whereas with moderate or no inconsistency the estimator from the MTC consistency model and the netmeta estimator showed acceptable properties. We also saw a dependency on the amount of heterogeneity. Concerning the evaluated methods to evaluate the consistency assumption, none was shown to be suitable.

**Conclusions:**

Based on our results we recommend a pragmatic approach for practical application in NMA. The estimator from the netmeta approach or the estimator from the Bayesian MTC consistency model should be preferred. Since none of the methods to evaluate the consistency assumption showed satisfactory results, users should have a strong focus on the similarity as well as the homogeneity assumption.

## Background

The combination of the results of several studies comparing the same two interventions is known as meta-analysis. The concept of meta-analysis and the corresponding methods are well established in medical statistics. However, in the recent years new methods for indirect comparisons have become more and more popular [1, 2]. These comprise both the adjusted indirect comparison of two interventions, which have not been compared directly in a head-to-head-trial, and the simultaneous comparison of more than two interventions in a network of interventions.

For all indirect comparisons there are three central assumptions. If there is any indication, that these assumptions are violated, no indirect comparison should be carried out at all. The homogeneity assumption is the same as for pairwise meta-analysis. There are already established methods to evaluate this assumption. Second, the similarity assumption, implies that all analyzed studies should be comparable (similar) regarding possible effect modifiers across all interventions. This is a qualitative assumption. Methods to evaluate this assumption will always have subjective components, so an objective evaluation will be difficult. There exist some detailed proposals for the evaluation of this assumption, for example by Cope et al. [3]. The assumption of consistency states that the effect estimations from direct and indirect evidence are consistent, meaning that there is no discrepancy between the results of direct and indirect comparisons (that cannot be explained by random error or heterogeneity) [4]. This assumption applies especially for indirect comparisons, which is why new statistical methods to evaluate this assumption have recently been developed or are still under development. However, little research has yet been conducted on their performance.

For simple networks like triangular networks with 3 interventions adjusted indirect comparisons [5] can be conducted. For more complex networks, simultaneous analysis of direct and indirect evidence as well as adequate inclusion of multi-arm studies, a network meta-analysis (NMA) is required. Thus, NMA is becoming increasingly popular in systematic reviews and health technology assessments [6, 7]. However, as well as for methods to evaluate the consistency assumption, there is still ambiguity concerning the properties of effect estimators in NMA. Several unsolved methodological problems [8] lead to a general uncertainty regarding the use and the certainty of results. Moreover, there are no established standards for the practical application.

Therefore we conducted a simulation study. The aim of our simulation study was to investigate the performance of effect estimators in NMA and the evaluation of the consistency assumption. While some simulation studies on NMA already exist, to our knowledge there are hardly any simulation studies analysing complex networks with up to 5 interventions. We also evaluated recently published effect estimators, not evaluated in a simulation study yet, as well as methods to evaluate the consistency assumption in complex networks. On the base of our results, we give recommendations for practical application. This paper is based on a PhD thesis, which includes all details [9]. An electronic version (in German) is available on request.

This paper is organized as follows. In the “Methods” section, we describe the different estimation approaches and methods to evaluate the consistency assumption. The design of our simulation study is described in the following Section. Subsequently the results of the simulation study are presented and illustrated by an application of a real data example. The paper will be closed with a discussion.

## Methods

The properties of the following effect estimators as well as methods for evaluating the consistency assumption were investigated in our simulation study.

### Effect estimators

Many methods have been proposed for effect estimation in NMA. We focused our investigation on effect estimators for NMA, which can be applied to all kinds of networks and which can handle multi-arm studies properly. Bayesian methods, often called mixed treatment comparisons (MTC), are most commonly used. But recently there has also been a strong focus on frequentist methods. We chose 3 different NMA estimators for our investigation (2 Bayesian, 1 frequentist), which will be described in more detail in the following Sections.

For comparison, we also included 3 direct effect estimators from pairwise meta-analysis. The first one is the frequentist DerSimonian-Laird meta-analysis estimator [10]. Although its shortcomings are now well known, especially with few studies [11], it is still the most frequently used estimator in meta-analysis. It is therefore computed for the purpose of comparison. The second one is the Bayesian meta-analysis estimator [12]. In the following, these estimators will be referred to as DE_Frequ_ and DE_Bayes_ respectively. We also had a look at the estimates from a so called MTC inconsistency model, where no consistency is assumed. Because it is closely related to the MTC consistency effect estimator, the MTC inconsistency model is described in more detail in the “MTC consistency model (MTC_Con_)” section.

For each estimator (direct and NMA) we fitted random effects (consistency) models assuming the same heterogeneity *τ*^2^ within each pairwise comparison in the network.

#### Graph-theoretical approach (netmeta)

The graph-theoretical approach is a frequentist method, developed by Rücker [13]. Methods from graph theory usually used in electrical networks were transferred to NMA. Briefly, for a network of *n* interventions and *m* pairwise comparisons from direct studies a *m*×*n* design matrix *B* is defined. Let *x*_*k*_ (*k*=1,…,*m*) denote the observed effects and *V*_*k*_ the corresponding variances. Then the *m*×*m* diagonal matrix *W* contains the inverse variances $\frac {1}{V_{k}}$. With the help of these two matrices a hat matrix *H* can be estimated by

$$ H = B \left(B^{T}WB \right)^{+} B^{T} W, $$

whereas (*B*^*T*^*WB*)^+^ is the Moore-Penrose pseudoinverse of the Laplacian matrix *L*=*B*^*T*^*WB*. Finally, by applying *H* to the vector of observed effects *x* consistent weighted least squares effect estimates $\hat {x}_{nma}$ are established.

As part of the implementation into the R-package *netmeta* [14] the originally fixed effect model was extend to a random effects model. The handling of multi-arm studies is described by Rücker und Schwarzer [15]. For the whole model and a more detailed description see also chapter 8 of Schwarzer et al. [16]. In the present article we will refer to this estimator as netmeta.

#### MTC consistency model (MTC_Con_)

The Bayesian MTC consistency model was first introduced by Lu and Ades [17, 18]. It is a hierarchical model, that combines direct and indirect evidence assuming consistency within a Bayesian framework. Suppose that there *n* interventions *A*,*B*,*C*,… to be compared in a network. A reference intervention has to be chosen, here denoted as *A*. The effects *d*_*Al*_ of all other interventions *l*=*B*,*C*,… with respect to *A* are modeled directly as basic parameters. Assuming consistency within the network, the effects of all other interventions can then be calculated by *d*_*bl*_=*d*_*Al*_−*d*_*Ab*_ for *l*≠*b*, *b*,*l*∈{*B*,*C*,…} as functional parameters.

For a binary outcome of study *k*, outcome counts for intervention *l* are summarized by the number of events *r*_*kl*_ out of a number *q*_*kl*_ of patients at risk. The number *r*_*kl*_ is assumed to follow a binomial distribution with parameters *p*_*kl*_ and *q*_*kl*_, whereas *p*_*kl*_ is modeled by a logit function. For each study *j*, a study specific baseline log-odds *μ*_*kb*_ of reference intervention *b* is assumed together with the log-odds ratio *δ*_*kbl*_ of the outcome for intervention *l* relative to this study specific reference *b*:

$$\begin{array}{@{}rcl@{}} r_{kl} & \sim & Bin\left(p_{kl}, q_{kl} \right)\\ \text{logit}\left(p_{kl} \right) & = &\begin{cases} \mu_{kb} \; \; \; \; \; \; \; \; \; \; \; \; \; \; b=A, B, C, \ldots \; \text{if} \; l=b\\ \mu_{kb} + \delta_{kbl} \; \; \; \; b=B, C, D, \ldots \; \text{if} \; l \; \text{before}\; b \; \text{(in alphabetical order)} \end{cases}. \end{array} $$

In a random effects model we assume the trial-specific *δ*_*kbl*_∼*N*(*d*_*bl*_,*τ*^2^) to follow a normal distribution with mean log-odds ratio *d*_*bl*_=*d*_*Al*_−*d*_*Ab*_ and homogeneous variance *τ*^2^. For multi-armed trials we consider a multivariate normal distribution with covariance $\frac {\tau ^{2}}{2}$ reflecting the assumption of homogeneous variance for all arms. For *μ*_*kb*_,*d*_*bl*_ and *τ*^2^ priors have to be established. Due to the lack of prior information we choose non informative priors. The exact specification as well as the initial values of Markov chains can be found in the Online Appendix [see Additional file 1]. For more information as well as exemplary WinBUGS code see Dias et al. [19]. In the present article, we will refer to this estimator as MTC_Con_.

As well as a consistency model (MTC_Con_), an inconsistency model can be fitted. Here, each of the mean relative effects *d*_*bl*_ is modelled separately. No consistency is assumed and hence no indirect evidence used. Therefore, this estimator is more a direct estimator than an NMA estimator and we will refer to it as an direct estimator. Only the variance *τ*^2^ will be estimated by all studies in the network collectively instead by one direct comparison alone [20]. In the following we will refer to it as MTC_Incon_.

#### MTC consistency model with stepwise removal of studies contributing to inconsistency identified in a leverage plot (MTC_SR_)

The second Bayesian estimator is also based on the MTC consistency model. Here, all inconsistent studies identified in a leverage plot are removed from the network in a stepwise procedure [21] finally leading to a consistent network. Using the residual deviance approach [22] the study (or study arm for multi-arm studies) contributing most to inconsistency according to the sum of the residual deviance and the leverage, will be eliminated from the analysis and the MTC consistency model will be recalculated. This process is repeated until the network demonstrates no more inconsistency (residual deviance + leverage ≤3) [23]. In the present article we will refer to this estimator as MTC_SR_.

### Evaluating the consistency assumption

Beside NMA effect estimators, we assessed the corresponding global methods for evaluating the consistency assumption described in the following.

#### *Q* statistic from graph-theoretical approach

The graph-theoretical approach enables the calculation of *Q* statistics and corresponding *I*^2^ for the whole network. The extent of variation in the whole network is measured by

$$Q_{total} = \left(x - \hat{x}_{nma} \right)^{T} W \left(x - \hat{x}_{nma} \right). $$

Under the assumptions of homogeneity and consistency *Q*_*total*_ follows a *χ*^2^ distribution with *M*−(*n*−1) degrees of freedom (df), where *M* denotes the number of independent studies in the network and *n* the number of interventions. *Q*_*total*_ can be decomposed into the sum of *k*=1,…,*K* statistics for heterogeneity between studies with the same design (set of treatments) in the network $\sum _{k=1}^{K} Q_{het_{k}}$ and the remaining design inconsistency *Q*_*incon*_. So *Q*_*incon*_ can be calculated by

$$Q_{incon} = Q_{total} - \sum_{k=1}^{K} Q_{het_{k}} $$

and follows a *χ*^2^ distribution with *K*−(*n*−1) degrees of freedom. For our simulation study we tested both *Q*_*total*_ and *Q*_*incon*_ with a level of significance of 0.2. We also calculated the corresponding $I^{2}_{total}$ and $I^{2}_{incon}$ by $I^{2} = \left (\frac {Q-df}{Q} \right) \times 100\%$ and assumed inconsistency if *I*^2^>50*%*.

#### Comparison of MTC consistency and MTC inconsistency model

We also compared the model fit of the MTC consistency model with an MTC inconsistency model. To assess model fit we used the residual deviance Dev _*res*_ as well as the deviance information criterion DIC [20, 24]. Using the residual deviance we assumed inconsistency if Dev _*res*_ from the inconsistency model was lower than Dev _*res*_ from the consistency model. For the DIC we introduced an additional threshold for relevance of 3 [24]. So we only assumed inconsistency if the DIC of the consistency model was more the 3 points higher than the DIC of the inconsistency model.

#### Stepwise removal of studies contributing to inconsistency identified in a leverage plot

By means of MTC_SR_ we assumed inconsistency when at least one study or study arm was excluded from the network. In the following we will refer to this approach as SR_Lev_.

## Simulation study

### Simulation scenarios

**Fig. 1:**
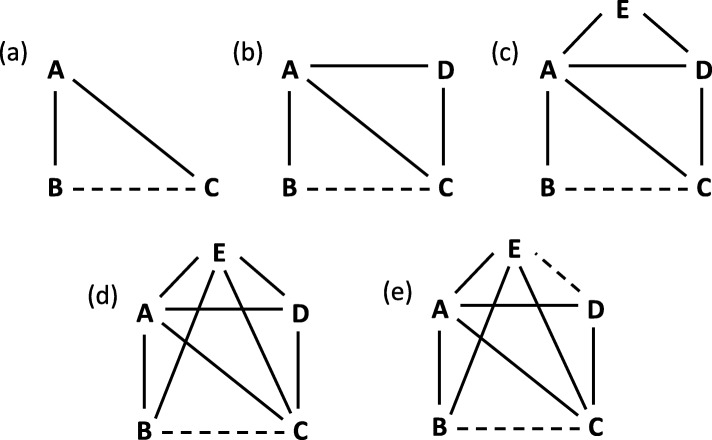
Graphics of simulated networks. Straight lines indicate direct evidence, dashed lines indicate indirect comparisons with potential inconsistency

We simulated data for 5 different kinds of network sizes and shapes, which are presented in Fig. 1. The straight lines in Fig. 1 indicate direct evidence, whereas the dashed lines indicate the comparison, where inconsistency was introduced in our simulations. We started with a triangular network (a) with 3 interventions (*A*,*B*,*C*) and data for each pairwise comparison. For network (b) we added an intervention *D* with direct comparisons to intervention *A* and *C*, but no direct comparisons to intervention *B*. For network (c) we again added another intervention *E* with direct comparisons to interventions *A* and *D*. From network (c) to network (d) we did not change the number of interventions, but we added more directs comparisons for intervention *E* with interventions *B* and *C* to the network. In the last network (e) we added an additional inconsistency for comparison *D* vs. *E* (*ROR*_*DE*_=0.6).

In data generation, we introduced inconsistency in the simulated networks by multiplying the consistent odds ratio (*OR*) with an ratio of odds ratios (*ROR*), i.e. for comparison *B* vs. *C*:

$$\text{OR}_{BC}^{incon} = \text{OR}_{BC} \times \text{ROR}_{BC}. $$

We set a *ROR* of 1 for no inconsistency, of 0.8 for moderate inconsistency and 0.6 for severe inconsistency. We also simulated a common heterogeneity between the study results in all direct comparisons. To avoid a too strong violation of the homogeneity assumption, we varied heterogeneity by a very small amount only. For a very low degree of heterogeneity we chose *τ*^2^ to be 0.01 and for a low degree of heterogeneity we chose 0.1.

Because of the high computational effort of Bayesian approaches we kept all others parameters fixed. We simulated data of 5 studies for each direct comparison in the network where each study arm contained 100 patients. For the binary endpoint we chose on *OR* as effect measure with a true treatment effect of 1 in all pairwise comparisons. The baseline risk for intervention *A* was set to be 0.1, all simulated studies were 2-arm studies. For each scenario we conducted *R*=1000 replications. An overview of all simulation input parameters is given in Table 1.

**Table 1:** Overview of simulated scenarios

**Networks**	(interventions, direct comparisons)
Network (a)	(*A*, *B*, *C*), 3
Network (b)	(*A*, *B*, *C*, *D*), 5
Network (c)	(*A*, *B*, *C*, *D*, *E*), 7
Network (d)	(*A*, *B*, *C*, *D*, *E*), 9
Network (e)	(*A*, *B*, *C*, *D*, *E*), 9, additional inconsistency for comparison *D* vs. *E*
**Inconsistency** ($\text {OR}_{BC}^{incon} = \text {OR}_{BC} \times \text {ROR}_{BC}$)	
No inconsistency	ROR_*BC*_=1
Moderate inconsistency	ROR_*BC*_=0.8
Severe inconsistency	ROR_*BC*_=0.6
**Heterogeneity**	
Very low heterogeneity	*τ*^2^=0.01
Low heterogeneity	*τ*^2^=0.1
Direct studies per pairwise comparison	*k*=5
Patients per study arm	*n*=100
True treatment effects	*OR*_*AB*_=…=*OR*_*DE*_=1.0
Baseline probability	*p*_*A*_=0.1
Replications	*R*=1000

### Generation of simulated data

In the following we will describe the generation of the data for network (d). For all other networks (a) to (c) we simply deleted all not required data leading to the desired network structure. The additional inconsistency in network (e) was similarly to the inconsistency in network (d).

For each pairwise comparison we drew *i*=1,…,*k* study-specific log-odds ratios *Y*_*ixy*_ from a normal distribution with mean *log*(*OR*_*xy*_) (*x*∈{*A*,*B*,*C*,*D*},*y*∈{*B*,*C*,*D*,*E*},*x*≠*y*) and variance *τ*^2^. For the inconsistent comparison *B* vs. *C* we used the mean $log(OR_{BC}^{incon})$. Using the study-specific *Y*_*ixy*_ and the true baseline probabilities *p*_*A*_ and $p_{B} = \frac {p_{A} \times OR_{AB}}{1-p_{A} \times \left (1-OR_{AB} \right)}$ we calculated the *i*=1,…,*k* study-specific baseline probabilities:

$${}{10pt}\begin{aligned} p_{i_{A}}=p_{A}, p_{i_{B}}&=\frac{p_{A} \times \exp \left(Y_{i_{AB}}\right)}{1 - p_{A} \times \left (1 - \exp \left(Y_{i_{AB}}\right) \right)}, \ldots, p_{i_{E}}=\frac{p_{A} \times \exp \left(Y_{i_{AE}}\right)}{1 - p_{A} \times \left (1 - \exp \left(Y_{i_{AE}}\right) \right)} \\ \text{and} \; p_{i_{C}}^{incon}&=\frac{p_{B} \times \exp \left(Y_{i_{BC}}^{incon}\right)}{1 - p_{B} \times \left (1 - \exp \left(Y_{i_{BC}}^{incon}\right) \right)}. \end{aligned} $$

For each study arm the number of events *r*_*X*_ was randomly generated assuming a binomial distribution with parameters *n* and $p_{i_{X}}$ (*X*∈{*A*,…,*E*}). For all pairwise comparisons including intervention *C* without inconsistency $p_{i_{C}}$ was used. For the simulated scenarios where inconsistency was introduced for comparison *B* vs. *C*, $p_{i_{C}}^{incon}$ was used. If the simulated number of events was 0 in a study arm, we added 0.5 to the cells of the corresponding 2×2 table.

### Performance

To evaluate the properties of the effect estimators we estimated the coverage probability (CP) of the 95% confidence or credible intervals by recording the percentage of replications where intervals included the true treatment effect. We also estimated the mean squared error (MSE) by

$$\widehat{\text{MSE}}(\hat{\theta})=\frac{1}{R} \times \sum_{j=1}^{R} \left (\hat{\theta}_{j} - \theta \right)^{2},  $$

with *θ* denoting the true parameter value and $\hat {\theta }_{j}$ the estimated value from replication *j* (*j*=1,…,*R*).

To ease interpretation of results we introduced a classification for the CP represented by a color coding. We classified a CP as good, if it was ∈[94*%*;96*%*] and as acceptable if it was ∈[90*%*;94*%*)∨(96*%*;100*%*]. A CP below 90*%* was classified as not acceptable. We also marked the estimators with the smallest MSE and the second smallest MSE by two or one stars. The actual values of the MSE for all effect estimators can be found in the Online Appendix [see Additional file 1]. An estimator with good properties should optimize the MSE under the side condition of an adequate CP.

For the methods to evaluate the consistency assumption we calculated the percentage of the correct and false decisions for inconsistency.

Again, we introduced a color coding to ease interpretation of results. In the cases, where inconsistency was present in our simulated data sets, we set the proportion of replications, in which a good approach should identify inconsistency to at least 90%. We categorized an approach as acceptable if this was the case in at least 75% of the replications. Below 75% we categorized the properties of the approach as not acceptable anymore. In the cases with no inconsistency in the data set, we set the cut-offs for good approach by a maximum of 5% of replications with identified inconsistency, an acceptable approach by a maximum of 25% and with more than 25% we categorized it as not acceptable anymore.

### Software implementation

We run the simulation study in the freely available software R 2.14.1 [25]. For the frequentist DerSimonian-Laird meta-analysis estimator we used the R package *metafor* (version 1.6-0) [26]. We implemented the Bayesian models by Markov chain Monte Carlo (MCMC) methodology into OpenBUGS (version 3.0.3) [27], which we called from R with the package BRugs (version 0.5-3) [28] using the example code provided by Dias et al. [19]. For each replication we used 3 chains with a burn-in of 20 000 iterations followed by 40 000 updates to obtain posterior estimates. Convergence was assessed by the Brooks-Gelman-Rubin method [29] and by visual inspection of the history plots of random samples. For trial baselines and basic parameters vague priors were specified.

The estimations for the graph-theoretical approach were calculated with the R package *netmeta* (version 0.3-1), which required a newer R version 3.0.2 [30]. The OpenBUGS code for the Bayesian models including the specification of the non informative prior distributions and the starting values for the 3 Markov chains is given in the Online Appendix [see Additional file 1].

## Results

For the evaluated effect estimators as well as the methods to evaluate the consistency assumption we focused our analysis on the 3 comparisons, which were available in all simulated networks: comparison *B* vs. *C*, where inconsistency was introduced, and the comparisons *A* vs. *B* and *A* vs. *C*. Together, all 3 form the closed loop *ABC*.

### Effect estimators

Table 2 shows the CP for all evaluated effect estimators and scenarios for comparison *B* vs. *C* that includes inconsistency in the simulated networks.

**Table 2:**
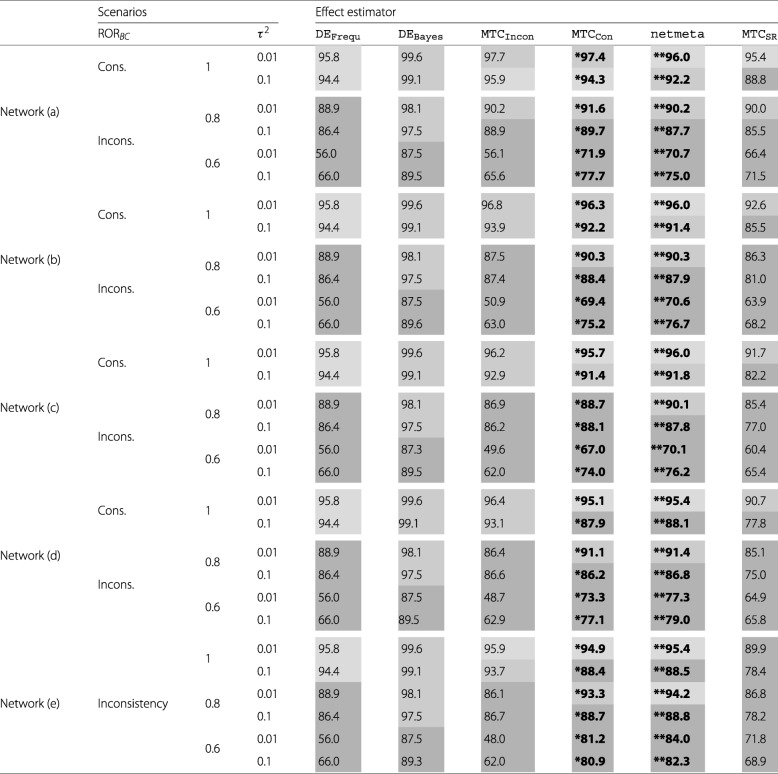
Combination of estimated coverage probabilities [in *%*] and MSE for comparison *B* vs. *C*

Coding:

^**^Smallest MSE

^*^Second smallest MSE

The first obvious result of Table 2 is, that in scenarios with severe inconsistency (*ROR*_*BC*_=0.6) non of the evaluated effect estimators showed an acceptable CP for comparison *B* vs. *C*. DE_Frequ_ showed good CP for the scenarios with no inconsistency, but no acceptable CP for the scenarios with a moderate degree of inconsistency. The CP of DE_Bayes_ for these scenarios was acceptable but always too high with values between 97.5*%* and 99.6*%*. MTC_Incon_ had no acceptable CP for all but one of the scenarios with moderate inconsistency. For the consistent scenarios its CP was at least acceptable, but slightly worse than the one from DE_Frequ_. Out of all the NMA estimators MTC_SR_ showed the worst CP for all scenarios. The two remaining NMA estimators MTC_Con_ and netmeta had a not acceptable CP in the case of moderate inconsistency and low heterogeneity. With moderate inconsistency and very low inconsistency however both showed an acceptable CP with one exception for MTC_Con_. In the consistent scenarios MTC_Con_ and netmeta had both at least acceptable CP with the exception of network (d) with low heterogeneity, where the CP for both estimators was not acceptable anymore. Concerning the MSE netmeta showed the smallest MSE for all scenarios, whereas MTC_Con_ had always the second smallest MSE.

The CP and the MSE for the comparisons *A* vs. *B* and *A* vs. *C* can be found in the Online Appendix [see Additional file 1]. As for these comparisons no inconsistency was introduced, all direct estimators showed at least acceptable coverage probabilities as it was to be expected. However, the network estimators use the information from the potential inconsistent comparison *B* vs. *C* also for these comparisons. Therefore their results are more interesting. For most of the simulated scenarios MTC_Con_ as well as netmeta showed acceptable and often even good CP. All exceptions for both estimators lay in the scenarios with severe inconsistency. MTC_SR_ however showed not acceptable CP in most of the scenarios. Also for the two comparisons *A* vs. *B* and *A* vs. *C*netmeta had the smallest and MTC_Con_ the second smallest MSE with few exceptions.

Additionally the simulation study showed a low dependency of the properties of the effect estimators on the network size. Especially for NMA estimators, validity of the homogeneity assumption is central, therefore its verification is crucial. Inclusion of additional studies is to be preferred over the inclusion of additional interventions.

### Evaluating the consistency assumption

Table 3 presents the results of the methods for evaluating the consistency assumption.

**Table 3:**
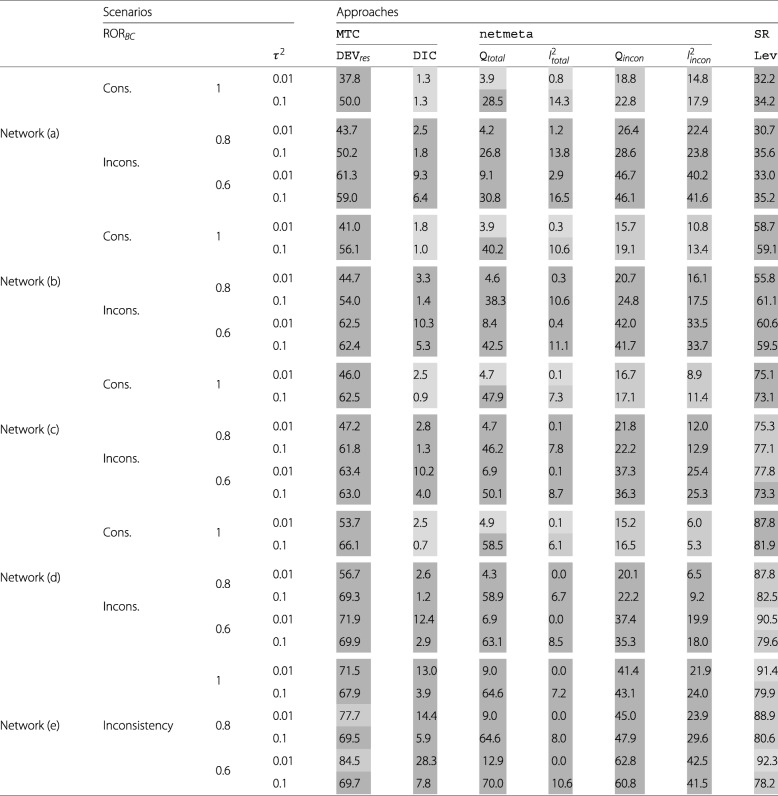
Proportion of replications with a decision for inconsistency [in *%*]

Coding with consistency:

Coding with inconsistency:

The proportion of replications with a wrong decision for inconsistency for Dev _*res*_ and SR_Lev_ was not acceptable with values ranging from 37.8% to 66.1% and from 32.2% to 87.8% respectively. *Q*_*incon*_ and $I^{2}_{incon}$ showed slightly better results under consistency with values between 15.2% - 22.8% and 5.3% - 17.9%, which we categorized as acceptable. Concerning the wrong decision for inconsistency *Q*_*total*_ and $I^{2}_{total}$ were highly dependable on the underlying heterogeneity. With very low heterogeneity in the networks (*τ*^2^=0.01) both showed low proportions of wrong decisions (≤5*%*), but with low heterogeneity (*τ*^2^=0.1) in the networks $I^{2}_{total}$ led to wrong decisions in 6.1% to 14.3% of the replications and *Q*_*total*_ in 28.5% to 58.5%. Only DIC showed good properties concerning the proportions of wrong decisions for inconsistency with only 0.7% to 2.5% in all scenarios with consistency. However, in the scenarios, where inconsistency was present, DIC indicated inconsistency only in a few replications as well (1.2% to 28.3%), which we categorized as not acceptable. Dev _*res*_ already showed high proportions of decisions for inconsistency in the scenarios with consistency, these values increased just slightly for the scenarios with inconsistency (43.7% to 84.5%) and were categorized as acceptable in only 2 scenarios (network (e), very low heterogeneity, moderate and severe inconsistency). All 4 methods for evaluating the consistency based on netmeta showed no acceptable proportions of decisions for inconsistency in any of the simulated scenarios with inconsistency. The values for *Q*_*total*_ ranged between 4.2% and 70.0% and for $I^{2}_{total}$ between 0% and 16.5%. It is remarkable however, that both methods showed noticeably higher proportions in the scenarios with low heterogeneity than the corresponding ones with very low heterogeneity. *Q*_*incon*_ and $I^{2}_{incon}$ indicated inconsistency in 20.1% to 62.8% and 6.5% to 42.5% of the replications for the scenarios with inconsistency respectively. SR_Lev_ already showed the highest proportions of decisions for inconsistency in the scenarios with consistency. These proportions increased, when inconsistency was present to values between 30.7% and 92.3%. With one exception (network (c), very low heterogeneity, severe inconsistency) these proportions were categorized as at least acceptable for the bigger networks (c), (d) and (e).

Overall we found that none of the evaluated methods reliably identified inconsistency and the identification of inconsistency just slightly depended of true underlying inconsistency. For some methods like the comparison of the consistency and the inconsistency MTC model by means of the Dev _*res*_ and the stepwise removal of studies contributing to inconsistency identified by a leverage plot the proportion of identified inconsistencies was relatively high. In contrast, for most methods, this proportion was rather small, independently to the underlying truth.

## Real data example

To illustrate the application of the different effect estimators as well as methods for evaluating the consistency assumption we applied them to a real data example.

**Fig. 2:**
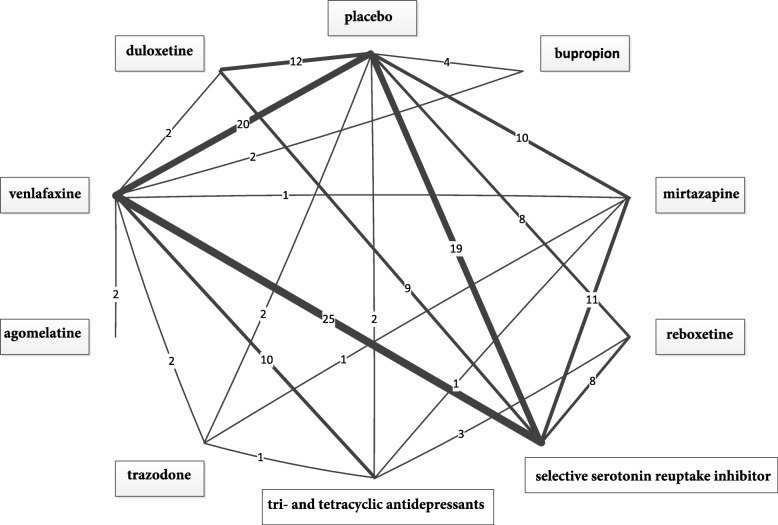
Network for response in the antidepressants example. The line width is proportional to the number of the underlying studies, the exact number of studies is also given

The data example is based on two previous benefit assessments for the treatment of depression carried out by the Institute for Quality and Efficiency in Health Care (IQWiG) [31, 32], which was also used by Sturtz and Bender [23]. We used the results for the outcome response to treatment. We had data for 7 different individual drugs, 2 drug classes and placebo. Because the data are taken from two previous benefit assessments it can be assumed that the similarity assumption is met at least roughly. We abandoned further investigation of this assumption because the objective of this example was purely methodological and no clinical conclusions should be made. The evaluation of the homogeneity assumption led to a exclusion of 3 two-arm studies and 1 arm from a three-arm study. This led to a final data pool of 100 studies (75 two-arm studies, 25 three-arm studies) for the comparison of the 10 treatments. For the possible 45 pairwise comparisons between all treatments, there were head-to-head studies for 21 of them. The network is shown in Fig. 2.

### Effect estimators

The results for the different evaluated effect estimators for the antidepressants network are presented in the Online Appendix [see Additional file 1]. Overall the results from netmeta and MTC_Con_ were very similar. With the exception of one comparison (tri- and tetracyclic antidepressants vs. placebo) both estimates agreed in statistical significance, point estimates and confidence/credible intervals. However, MTC_SR_ provided much more frequently statistically significant results. The direct estimator DE_Bayes_ turned out to be not suitable due to very wide credible intervals. Additionally, the results illustrated one of the main advantages of network meta analysis, which was not as obvious in the simulation study. While the direct estimators can only provide effect estimates if head to head studies are available, network meta analysis estimators provide estimates for all possible comparisons in the network.

### Evaluating the consistency assumption

For evaluating the consistency assumption by comparing the MTC consistency and the MTC inconsistency model the two models were fit to the data and the DIC and the residual deviance were calculated. The DIC was 373.1 for the inconsistency model and 370.1 for the consistency model whereas the residual deviance was 244.5 for the inconsistency model and 237.7 for the consistency model. Thus both measures for model fit favored the consistency model and identified no sign of global inconsistency. (It should be noted that, with 225 data points in the network the consistency model had not an optimal model fit as well.) Likewise the different evaluated methods for evaluating the consistency assumption from the software package netmeta were applied to the data example. For $\texttt {I}^{2}_{total}$ and $\texttt {I}^{2}_{incon}$ this resulted in values of 23.5*%* and 8.3*%*. Comparing them with a cut-off of 50*%*, both statistics showed no global inconsistency. The same holds for Q_*incon*_. The corresponding p-value was 0.637 and hence not statistically significant at a predefined level of significance of 0.2. Only Q_*total*_ identified global inconsistency with a corresponding p-value of 0.015. MTC_SR_ identified inconsistency in the network with a total of 4 inconsistent 2-arm studies and 4 inconsistent arms of 3-arm studies. In Table 4 an overview of the results of the different methods to evaluate the consistency assumption in the antidepressants example is given.

**Table 4:** Overview of the identification of global inconsistency in the antidepressants example for all evaluated methods

MTC		netmeta				SR
DEV_*res*_	DIC	Q_*total*_	I*total*2	Q_*incon*_	I*incon*2	Lev
−	−	+	−	−	−	+

^+^Global inconsistency

^-^No global inconsistency

The evaluated methods showed different results for the global inconsistency. Five methods identified no inconsistency, whereas two (Q_*total*_ and SR_Lev_) identified inconsistency.

## Discussion

In this paper, we presented a simulation study aimed to investigate the properties of different effect estimators and methods to evaluate the consistency assumption in NMA. The results of our study indicated that with moderate or no inconsistency and very low heterogeneity the estimator from a Bayesian MTC consistency model MTC_Con_ and the estimator from the graph-theoretical approach by the R-package netmeta showed acceptable properties concerning a coverage probability of 90*%* and higher and a relatively small MSE, whereas netmeta had slightly better properties. However, none of the evaluated effect estimators showed acceptable properties for networks with a high degree of inconsistency. Coverage probabilities for MTC_Con_ and netmeta ranged from 67.0% to 81.2% (MTC_Con_) and from 70.1% to 84.0% (netmeta), respectively. So, there is a strong need to evaluate and ensure the consistency assumption to get effect estimates with acceptable properties. If the consistency assumption is seriously violated, no NMA should be carried out at all. However, concerning the evaluated methods to evaluate the consistency assumption, none were shown to be suitable. This a huge problem for the application of NMA in practice. All evaluated methods showed either high or low proportions of replications with a decision for inconsistency in all scenarios independently of the underlying true consistency. The comparison of the MTC consistency and MTC inconsistency model by the residual deviance Dev_*res*_ (37.8%-84.5%) and the stepwise removal of studies contributing to inconsistency identified in a leverage plot MTC_SR_ (30.7%-92.3%) showed in all scenarios rather high proportions, which increased with network size. For the DIC the proportions were always small with values up to a maximum of 28.3% and slightly higher proportions in scenarios with very low heterogeneity. The methods based on the graph-theoretical approach measuring the extent of the variation in the whole network *Q*_*total*_ and $I^{2}_{total}$ showed higher proportions of decisions for inconsistency in all scenarios with low heterogeneity. This was particularly pronounced for *Q*_*total*_. For example in network (d) with a high degree of inconsistency and very low heterogeneity there were only 6.9% correct decisions for inconsistency, but with low heterogeneity this proportion increased to 63.1% even though the same amount of inconsistency was present in the generated data set. The findings suggest, that these methods detect rather heterogeneity not inconsistency. The methods based on the graph-theoretical approach measuring the extent of the variation in the network caused by inconsistency *Q*_*incon*_ and $I^{2}_{incon}$ both led to acceptable proportions of wrong decisions for inconsistency with values between 15.2% and 22.8% and 5.3% and 17.9% respectively. However, in the scenarios with underlying inconsistency both methods detected inconsistency only in a maximum of 62.8% of the replications. This means that the proportion of detected inconsistencies did just slightly differ between the different scenarios with or without true inconsistency for all methods. We also saw a dependency to the amount of heterogeneity and only a low impact of the network size, whereas better properties for a network with more studies than with more interventions were observed.

Different aspects of the properties of effect estimators and methods to evaluate the consistency assumption in NMA have also been evaluated in other simulation studies [33–38]. However, most of the existing simulation studies evaluated the adjusted indirect comparison according to Bucher [5]. Song et al. [36] were the first ones, who also evaluated the Bayesian MTC effect estimator as well as methods to evaluate the consistency assumption. Similar to our study, they found that all effect estimators provided unbiased results, when no inconsistency was present. For the methods to evaluate the consistency assumption the power to detect inconsistencies was very small. Even with 120 studies the maximum power was about 70%. Jonas et al. [37] evaluated the properties of the Bayesian MTC effect estimator for the probability to be the best intervention in networks with up to 4 interventions and found only little influence of the number of studies (2 to 10) per pairwise comparison in the network. Veroniki et al. [38] evaluated the influence of different network properties on the estimation of inconsistency in a network of 3 interventions by the difference of the direct estimation and the adjusted indirect estimation according to Bucher [5]. As well as Song et al. [36] they found, that the test had low power, wheres the power was slightly higher when estimating the heterogeneity variance for the pairwise comparisons with the method by Knapp and Hartung [39, 40]. The low power was similar to the findings in our simulation study for the more complex methods to evaluate the consistency assumption for networks with up to 5 interventions.

Most simulation studies regarding NMA consider only networks with 4 or less interventions. We conducted a simulation study for networks with up to 5 interventions. Moreover the evaluated effect estimators for NMA in our simulation study differ from previous simulation studies with the majority evaluating the adjusted indirect comparison according to Bucher [5]. However, for more complex network structures this simple approach is not possible. The estimator netmeta as well as the estimator based on a MTC consistency model with stepwise removal of studies contributing to inconsistency identified in a leverage plot MTC_SR_ have never been evaluated before. Until now, there also have only been few studies, which evaluated methods to evaluate the consistency assumption at all and most of them compared direct and indirect evidence in a very simple way [36, 38]. Again, for more complex networks this approach is not possible.

Because of the computational intensity of the Bayesian MCMC methods, only a limited number of scenarios could be considered in this simulation study. In addition to the network size, consistency and heterogeneity, the numbers of studies per pairwise comparison, the sample size in the studies, the true underlying effects, the baseline probability and other effect measures would be interesting to assess. For ease of implementation only two-arm studies were simulated. Since all evaluated approaches can handle multi-arm studies properly, the impact of multi-arm studies should also be a topic of further research. In the simulated data as well as in the models underlying the NMA estimators the same heterogeneity *τ*^2^ was assumed in each pairwise comparison. This homogeneous variance structure has already been questioned by others and should be further evaluated. Thorlund et al. [41] for example suggest for the Bayesian context the use of informative priors instead.

Another result of our study is that definitely further evaluation of the approaches in NMA is needed. Especially reliable methods to evaluate the consistency assumption in complex networks are missing and future research should focus on that topic. We also evaluated only global methods to evaluate the consistency assumption. A further evaluation of local methods like node-splitting [22] and the newly proposed composite likelihood method [42] could be helpful in detecting inconsistency and dealing with it. Another way to deal with the current lack of reliable methods to evaluate the consistency assumption could be the use of estimators, which can handle a higher degree of inconsistency, like models with inconsistency parameters [43–48]. The properties of these estimators should also be investigated in further simulation studies. Furthermore it is a problem for the methods to evaluate the consistency assumption to distinguish between heterogeneity and inconsistency. This is another important topic, where more research is required.

After the implementation of this simulation study, there has been the development of a user-friendly R package *gemtc* [49] for conducting Bayesian network meta-anaylsis using *JAGS* (Just another Gibbs sampler) with several useful features. Users, more familiar with the software R, can use this package instead of the BUGS software. For a guide to the practical application of *gemtc* as well as *netmeta* see also [50].

## Conclusions

**Fig. 3:**
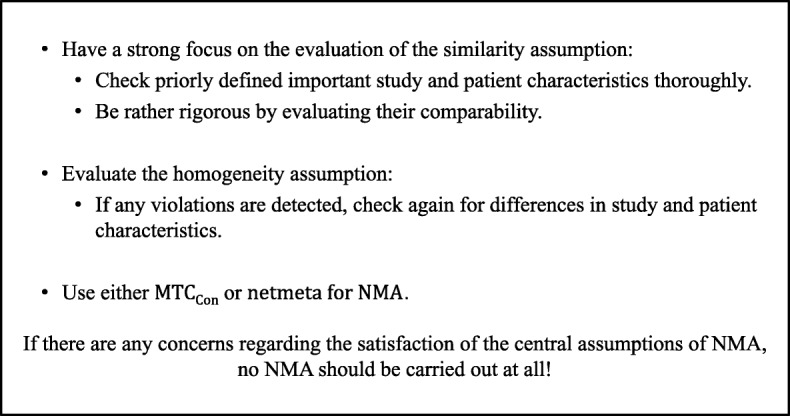
Recommended pragmatic approach

According to the results of our simulation study we recommend a pragmatic approach as currently best possible procedure for practical application in NMA, which is shown in Fig. 3. The estimators netmeta or MTC_Con_ showed the best properties concerning coverage probability and mean squared error and therefore should be used. Since none of the evaluated methods for checking the consistency assumption showed acceptable properties, there should be a strong focus on the evaluation of the similarity assumption and one should rather be rigorous by evaluating it, since it is currently the only way to avoid a high risk of inconsistency in the network as well. Important study and patient characteristics for the investigated research question should be defined a priori and the studies included in NMA should be comparable regarding these characteristics. It is also very important to evaluate the homogeneity assumption and make sure, there are no violations. If violations are detected by a statistical test for homogeneity, the studies should be checked again for differences in their characteristics that can potentially explain the heterogeneity. If there are any concerns regarding the satisfaction of these central assumptions of NMA, no NMA should be carried out at all. Additionally, networks with more studies over networks with more interventions should be preferred.

## Supplementary information

**Additional file 1** Online appendix. Additional tables and OpenBUGS Code for the Bayesian models including the specification of the non informative prior distributions.

## Data Availability

The data are available in Kiefer [9] via the German National Library of Medicine (ZB MED) in Cologne (Database: Catalogue ZB MED Medicine, Health; 38 M K: ZB MED, Shelf mark: 2016 D 392).

## Abbreviations

CP: Coverage probability
DE: Direct effect estimator
DE_Frequ_: Frequentist direct effect estimator
DE_Bayes_: Bayesian direct effect estimator
Dev _*res*_: Residual deviance
DIC: Deviance information criterion
Lev: Leverage
MCMC: Markov chain Monte Carlo
MSE: Mean squared error
MTC: Mixed treatment comparison
MTC_Con_: MTC consistency model
MTC_Incon_: MTC inconsistency model
MTC_SR_: MTC consistency model with stepwise removal of studies
NMA: Network meta-analysis
OR: Odds ratio
ROR: Ratio of odds ratios
SR_Lev_: Stepwise removal of studies by means of the leverage plot
