# Supplementary material for: A simulation study to compare different estimation approaches for network meta-analysis and corresponding methods to evaluate the consistency assumption
Source: BMC Med Res Methodol. 2020 Feb 24;20:36. doi: 10.1186/s12874-020-0917-3 (PMC7041240; doi:10.1186/s12874-020-0917-3)
Supplement: Supplementary file 1 — Additional file 1 Online appendix. Additional tables and OpenBUGS Code for the Bayesian models including the specification of the non informative prior distributions. [file 12874_2020_917_MOESM1_ESM.pdf]

Online Appendix

A simulation study to compare different estimation approaches for network meta-analysis and corresponding methods to evaluate the consistency assumption

C. Kiefer, S.Sturtz and R. Bender

August 06, 2019

1 Tables

Table 1: Combination of estimated coverage probabilities [in %] and MSE of the interval and point estimates for comparison A vs. B

		Scenarios		Effect estimator					
		ROR_{BC}	τ^2	$\mathtt{DE}_{\mathtt{Frequ}}$	$\mathtt{DE}_{\mathtt{Bayes}}$	$\mathtt{MTC}_{\mathtt{Incon}}$	$\mathtt{MTC}_{\mathtt{Con}}$	`netmeta`	$\mathtt{MTC}_{\mathtt{SR}}$
Network (a)	Cons.	1	0.01	97.3	99.8	97.6	***97.6**	****96.1**	95.5
			0.1	94.6	99.6	97.2	***94.7**	****92.5**	90.2
	Incons.	0.8	0.01	97.6	99.7	98.4	***96.3**	****94.8**	93.5
			0.1	***95.8**	99.6	97.3	93.6	****90.8**	91.2
		0.6	0.01	****96.6**	99.9	***97.8**	93.5	91.4	89.3
			0.1	****94.7**	99.6	***97.3**	88.9	86.4	85.3
Network (b)	Cons.	1	0.01	97.3	99.8	96.5	***96.6**	****95.7**	92.6
			0.1	94.6	99.6	96.4	***92.7**	****91.2**	87.5
	Incons.	0.8	0.01	97.6	99.7	97.5	***94.3**	****94.1**	89.7
			0.1	***95.8**	99.6	96.9	92.0	****90.5**	85.4
		0.6	0.01	****96.6**	99.9	***96.7**	89.6	88.7	84.3
			0.1	****94.7**	99.6	***96.7**	85.6	83.6	78.6
Network (c)	Cons.	1	0.01	97.3	99.8	96.3	***95.8**	****95.5**	90.7
			0.1	94.6	99.6	96.5	***92.4**	****91.7**	84.7
	Incons.	0.8	0.01	97.6	99.7	96.9	***93.5**	****94.4**	88.4
			0.1	***95.8**	99.6	96.4	90.9	****90.6**	82.5
		0.6	0.01	****96.6**	99.9	***96.1**	87.4	88.5	81.5
			0.1	****94.7**	99.6	***96.8**	83.2	83.7	76.0
Network (d)	Cons.	1	0.01	97.3	99.8	95.7	***95.2**	****95.9**	89.7
			0.1	94.6	99.6	96.0	***90.7**	****90.1**	81.4
	Incons.	0.8	0.01	97.6	99.7	96.4	***93.2**	****94.1**	87.8
			0.1	95.8	99.6	96.4	***91.3**	****90.7**	81.2
		0.6	0.01	96.6	99.9	95.4	***89.5**	****91.0**	82.2
			0.1	***94.7**	99.6	96.4	85.2	****84.7**	77.4
Network (e)	Inconsistency	1	0.01	97.3	99.8	96.0	***96.0**	****95.9**	88.6
			0.1	94.6	99.8	95.9	***91.3**	****91.5**	83.3
		0.8	0.01	97.6	99.7	96.3	***95.4**	****95.4**	89.2
			0.1	95.8	99.6	96.5	***93.7**	****93.3**	83.6
		0.6	0.01	96.6	99.9	95.3	***93.8**	****93.9**	85.9
			0.1	94.7	99.4	96.9	***88.3**	****87.3**	79.4

Coding: $\in [94\,\%; 96\,\%]$ | $\in [90\,\%; 94\,\%) \vee (96\,\%; 100\,\%]$ | $\in [0\,\%; 90\,\%)$

**: Smallest MSE; *: Second smallest MSE

Table 2: Combination of estimated coverage probabilities [in %] and MSE of the interval and point estimates for comparison A vs. C

Network	Scenarios	ROR_{BC}	τ^2	$\text{DE}_{\texttt{Frequ}}$	$\text{DE}_{\texttt{Bayes}}$	$\text{MTC}_{\texttt{Incon}}$	$\text{MTC}_{\texttt{Con}}$	`netmeta`	MTC_{SR}
Network (a)	Cons.	1	0.01	96.8	100.0	98.3	***97.5**	****96.5**	95.4
			0.1	95.8	99.6	97.9	***98.6**	****97.1**	94.6
	Incons.	0.8	0.01	96.5	99.7	97.8	***96.4**	****94.7**	94.5
			0.1	94.8	99.7	96.6	***97.5**	****96.5**	94.7
		0.6	0.01	****97.1**	99.9	***98.3**	91.1	88.4	87.1
			0.1	***92.8**	99.6	97.1	93.5	****92.3**	89.4
Network (b)	Cons.	1	0.01	96.8	100.0	97.1	***96.1**	****95.7**	91.4
			0.1	95.8	99.6	97.1	***96.2**	****95.7**	90.6
	Incons.	0.8	0.01	96.5	99.7	96.6	***95.1**	****95.3**	92.1
			0.1	94.8	99.7	96.4	***95.3**	****94.7**	90.8
		0.6	0.01	97.1	99.9	96.9	***91.6**	****90.8**	86.9
			0.1	92.8	99.6	95.9	***91.5**	****91.6**	85.3
Network (c)	Cons.	1	0.01	96.8	100.0	96.8	***95.6**	****95.6**	90.1
			0.1	95.8	99.6	96.9	***95.9**	****95.8**	87.7
	Incons.	0.8	0.01	96.5	99.7	96.4	***95.5**	****95.3**	89.4
			0.1	94.8	99.7	95.9	***95.3**	****95.4**	87.1
		0.6	0.01	97.1	99.9	96.7	***90.5**	****91.1**	84.6
			0.1	92.8	99.6	95.8	***92.0**	****91.4**	82.7
Network (d)	Cons.	1	0.01	96.8	100.0	96.7	***95.3**	****95.7**	89.1
			0.1	95.8	99.6	97.3	***95.2**	****95.1**	85.6
	Incons.	0.8	0.01	96.5	99.7	96.0	***95.1**	****95.5**	89.2
			0.1	94.8	99.7	95.8	***94.0**	****94.0**	85.3
		0.6	0.01	97.1	99.9	95.9	***90.5**	****91.6**	85.4
			0.1	92.8	99.6	96.4	***93.0**	****92.6**	81.3
Network (e)	Inconsistency	1	0.01	96.8	100.0	96.2	***95.4**	****95.6**	88.5
			0.1	95.8	99.6	97.4	***95.3**	****95.6**	86.5
		0.8	0.01	96.5	99.7	95.3	***95.4**	****95.6**	89.2
			0.1	94.8	99.7	96.2	***94.5**	****94.6**	86.1
		0.6	0.01	97.1	99.9	96.4	***91.0**	****92.0**	84.6
			0.1	92.8	99.4	96.8	***92.6**	****92.5**	82.0

Coding: $\in [94\,\%; 96\,\%]$ (light grey) $\in [90\,\%; 94\,\%) \vee (96\,\%; 100\,\%]$ (medium grey) $\in [0\,\%; 90\,\%)$ (dark grey)

**: Smallest MSE; *: Second smallest MSE

Table 3: Estimated MSE of effect estimates for comparison B vs. C

	Scenarios			Effect estimator					
		ROR_{BC}	τ^2	$\texttt{DE}_{\texttt{Frequ}}$	$\texttt{DE}_{\texttt{Bayes}}$	$\texttt{MTC}_{\texttt{Incon}}$	$\texttt{MTC}_{\texttt{Con}}$	`netmeta`	$\texttt{MTC}_{\texttt{SR}}$
Network (a)	Cons.	1	0.01	0.046	0.054	0.052	***0.034**	****0.030**	0.042
			0.1	0.086	0.104	0.097	***0.066**	****0.058**	0.081
	Incons.	0.8	0.01	0.100	0.117	0.112	***0.060**	****0.052**	0.069
			0.1	0.132	0.158	0.150	***0.087**	****0.075**	0.104
		0.6	0.01	0.308	0.376	0.353	***0.158**	****0.131**	0.173
			0.1	0.333	0.423	0.386	***0.188**	****0.151**	0.203
Network (b)	Cons.	1	0.01	0.046	0.054	0.050	***0.031**	****0.028**	0.042
			0.1	0.086	0.104	0.095	***0.063**	****0.055**	0.084
	Incons.	0.8	0.01	0.100	0.117	0.111	***0.055**	****0.047**	0.067
			0.1	0.132	0.158	0.149	***0.081**	****0.070**	0.104
		0.6	0.01	0.308	0.376	0.348	***0.136**	****0.113**	0.154
			0.1	0.333	0.422	0.382	***0.161**	****0.134**	0.197
Network (c)	Cons.	1	0.01	0.046	0.054	0.050	***0.031**	****0.028**	0.042
			0.1	0.086	0.104	0.095	***0.063**	****0.055**	0.091
	Incons.	0.8	0.01	0.100	0.117	0.109	***0.054**	****0.047**	0.068
			0.1	0.132	0.158	0.148	***0.079**	****0.069**	0.113
		0.6	0.01	0.308	0.380	0.340	***0.132**	****0.110**	0.157
			0.1	0.333	0.423	0.379	***0.158**	****0.132**	0.195
Network (d)	Cons.	1	0.01	0.046	0.054	0.048	***0.024**	****0.022**	0.033
			0.1	0.086	0.104	0.095	***0.055**	****0.048**	0.077
	Incons.	0.8	0.01	0.100	0.117	0.105	***0.036**	****0.032**	0.048
			0.1	0.132	0.158	0.147	***0.063**	****0.055**	0.087
		0.6	0.01	0.308	0.376	0.333	***0.081**	****0.068**	0.095
			0.1	0.333	0.423	0.378	***0.107**	****0.089**	0.132
Network (e)	Inconsistency	1	0.01	0.046	0.054	0.050	***0.025**	****0.023**	0.034
			0.1	0.086	0.102	0.095	***0.057**	****0.050**	0.078
		0.8	0.01	0.100	0.117	0.111	***0.032**	****0.028**	0.045
			0.1	0.132	0.158	0.147	***0.058**	****0.051**	0.084
		0.6	0.01	0.308	0.376	0.334	***0.067**	****0.056**	0.082
			0.1	0.333	0.425	0.379	***0.094**	****0.080**	0.126

**: Smallest MSE; *: Second smallest MSE

Table 4: Estimated MSE of effect estimates for comparison A vs. B

Scenarios		ROR_{BC}	τ^2	$\texttt{DE}_{\texttt{Frequ}}$	$\texttt{DE}_{\texttt{Bayes}}$	$\texttt{MTC}_{\texttt{Incon}}$	$\texttt{MTC}_{\texttt{Con}}$	`netmeta`	$\texttt{MTC}_{\texttt{SR}}$
Network (a)	Cons.	1	0.01	0.046	0.053	0.051	***0.035**	****0.031**	0.043
			0.1	0.064	0.076	0.073	***0.060**	****0.053**	0.072
	Incons.	0.8	0.01	0.046	0.055	0.052	***0.043**	****0.037**	0.051
			0.1	***0.059**	0.069	0.066	0.063	****0.056**	0.076
		0.6	0.01	****0.047**	0.053	***0.052**	0.062	0.053	0.073
			0.1	****0.064**	0.074	***0.071**	0.095	0.081	0.106
Network (b)	Cons.	1	0.01	0.046	0.053	0.050	***0.033**	****0.030**	0.044
			0.1	0.064	0.076	0.072	***0.059**	****0.053**	0.074
	Incons.	0.8	0.01	0.046	0.055	0.051	***0.042**	****0.037**	0.056
			0.1	***0.059**	0.069	0.065	0.064	****0.057**	0.085
		0.6	0.01	****0.047**	0.053	***0.050**	0.065	0.056	0.085
			0.1	****0.064**	0.074	***0.071**	0.098	0.087	0.130
Network (c)	Cons.	1	0.01	0.046	0.053	0.049	***0.032**	****0.029**	0.044
			0.1	0.064	0.076	0.072	***0.059**	****0.052**	0.078
	Incons.	0.8	0.01	0.046	0.055	0.050	***0.043**	****0.037**	0.057
			0.1	***0.059**	0.069	0.066	0.065	****0.057**	0.095
		0.6	0.01	****0.047**	0.053	***0.050**	0.067	0.056	0.090
			0.1	****0.064**	0.074	***0.071**	0.101	0.088	0.136
Network (d)	Cons.	1	0.01	0.046	0.053	0.048	***0.025**	****0.023**	0.035
			0.1	0.064	0.076	0.071	***0.048**	****0.043**	0.066
	Incons.	0.8	0.01	0.046	0.055	0.049	***0.030**	****0.026**	0.040
			0.1	0.059	0.069	0.065	***0.047**	****0.042**	0.067
		0.6	0.01	0.047	0.053	0.049	***0.040**	****0.034**	0.055
			0.1	***0.064**	0.074	0.070	0.070	****0.061**	0.088
Network (e)	Inconsistency	1	0.01	0.046	0.053	0.049	***0.027**	****0.024**	0.037
			0.1	0.064	0.074	0.071	***0.048**	****0.042**	0.064
		0.8	0.01	0.046	0.055	0.050	***0.028**	****0.024**	0.037
			0.1	0.059	0.069	0.065	***0.044**	****0.039**	0.063
		0.6	0.01	0.047	0.053	0.049	***0.033**	****0.028**	0.048
			0.1	0.064	0.076	0.071	***0.063**	****0.055**	0.082

**: Smallest MSE; *: Second smallest MSE

Table 5: Estimated MSE of effect estimates for comparison A vs. C

Scenarios		ROR_{BC}	τ^2	$\mathtt{DE}_{\mathtt{Frequ}}$	$\mathtt{DE}_{\mathtt{Bayes}}$	$\mathtt{MTC}_{\mathtt{Incon}}$	$\mathtt{MTC}_{\mathtt{Con}}$	netmeta	$\mathtt{MTC}_{\mathtt{SR}}$
Network (a)	Cons.	1	0.01	0.047	0.054	0.052	***0.034**	****0.030**	0.044
			0.1	0.062	0.074	0.070	***0.043**	****0.038**	0.055
	Incons.	0.8	0.01	0.047	0.054	0.051	***0.038**	****0.034**	0.048
			0.1	0.063	0.077	0.072	***0.049**	****0.041**	0.056
		0.6	0.01	****0.045**	0.053	***0.051**	0.066	0.057	0.079
			0.1	***0.068**	0.079	0.077	0.073	****0.060**	0.086
Network (b)	Cons.	1	0.01	0.047	0.054	0.051	***0.026**	****0.023**	0.036
			0.1	0.062	0.074	0.070	***0.036**	****0.032**	0.049
	Incons.	0.8	0.01	0.047	0.054	0.051	***0.028**	****0.025**	0.037
			0.1	0.063	0.077	0.071	***0.040**	****0.034**	0.049
		0.6	0.01	0.045	0.053	0.050	***0.042**	****0.036**	0.051
			0.1	0.068	0.080	0.078	***0.053**	****0.044**	0.068
Network (c)	Cons.	1	0.01	0.047	0.054	0.051	***0.024**	****0.022**	0.036
			0.1	0.062	0.074	0.069	***0.035**	****0.031**	0.052
	Incons.	0.8	0.01	0.047	0.054	0.051	***0.026**	****0.024**	0.039
			0.1	0.063	0.077	0.071	***0.037**	****0.033**	0.051
		0.6	0.01	0.045	0.053	0.049	***0.038**	****0.033**	0.051
			0.1	0.068	0.079	0.075	***0.050**	****0.042**	0.068
Network (d)	Cons.	1	0.01	0.047	0.054	0.049	***0.021**	****0.019**	0.030
			0.1	0.062	0.074	0.069	***0.032**	****0.028**	0.046
	Incons.	0.8	0.01	0.047	0.054	0.049	***0.022**	****0.019**	0.031
			0.1	0.063	0.077	0.070	***0.035**	****0.030**	0.048
		0.6	0.01	0.045	0.053	0.048	***0.030**	****0.026**	0.040
			0.1	0.068	0.079	0.075	***0.042**	****0.036**	0.057
Network (e)	Inconsistency	1	0.01	0.047	0.054	0.050	***0.021**	****0.019**	0.032
			0.1	0.062	0.074	0.068	***0.032**	****0.029**	0.046
		0.8	0.01	0.047	0.054	0.050	***0.023**	****0.020**	0.032
			0.1	0.063	0.077	0.070	***0.035**	****0.030**	0.046
		0.6	0.01	0.045	0.053	0.048	***0.030**	****0.026**	0.040
			0.1	0.068	0.079	0.075	***0.042**	****0.036**	0.057

**: Smallest MSE; *: Second smallest MSE

Table 6: Effect estimates of the evaluated effect estimators for the antidepressants data example

Comparison	N	N_I	$\texttt{DE}_{\texttt{Frequ}}$ OR [95 % − CI]	$\texttt{DE}_{\texttt{Bayes}}$ OR [95 % − CrI]	$\texttt{MTC}_{\texttt{Incon}}$ OR [95 % − CrI]	$\texttt{MTC}_{\texttt{Con}}$ OR [95 % − CrI]	netmeta OR [95 % − CI]	$\texttt{MTC}_{\texttt{SR}}$ OR [95 % − CrI]
A vs. P						**2.63 [1.57; 4.24]**	**2.55 [1.62; 4.02]**	**2.68 [1.79; 4.07]**
B vs. P	4	4	**1.48 [1.20; 1.82]**	**1.48 [1.04; 2.10]**	**1.48 [1.19; 1.84]**	**1.48 [1.16; 1.91]**	**1.46 [1.16; 1.85]**	**1.48 [1.24; 1.77]**
D vs. P	12	12	**1.99 [1.65; 2.39]**	**2.00 [1.62; 2.49]**	**1.95 [1.68; 2.27]**	**1.82 [1.55; 2.11]**	**1.79 [1.55; 2.06]**	**1.65 [1.41; 1.90]**
M vs. P	10	10	**1.87 [1.36; 2.58]**	**1.92 [1.32; 2.88]**	**1.88 [1.48; 2.41]**	**1.91 [1.61; 2.26]**	**1.87 [1.58; 2.22]**	**1.77 [1.48; 2.08]**
R vs. P	7	7	1.24 [0.98; 1.56]	1.24 [0.90; 1.71]	**1.25 [1.04; 1.48]**	**1.30 [1.10; 1.54]**	**1.28 [1.08; 1.52]**	**1.21 [1.05; 1.39]**
T vs. P	2	2	1.22 [0.72; 2.07]	1.21 [0.00; 328.98]	1.39 [0.84; 2.27]	1.14 [0.72; 1.81]	1.16 [0.77; 1.74]	1.19 [0.82; 1.65]
V vs. P	20	20	**2.04 [1.74; 2.38]**	**2.05 [1.74; 2.41]**	**2.08 [1.82; 2.34]**	**2.06 [1.83; 2.30]**	**2.03 [1.82; 2.27]**	**2.10 [1.88; 2.33]**
SSRI vs. P	19	19	**1.54 [1.32; 1.78]**	**1.54 [1.31; 1.82]**	**1.62 [1.43; 1.82]**	**1.69 [1.52; 1.88]**	**1.67 [1.50; 1.85]**	**1.62 [1.47; 1.78]**
TZA vs. P	1	1	1.17 [0.60; 2.28]	1.17 [0.60; 2.30]	1.02 [0.55; 1.92]	**2.18 [1.72; 2.76]**	**2.12 [1.62; 2.77]**	**2.53 [1.95; 3.25]**
B vs. A						**0.56 [0.34; 0.97]**	**0.57 [0.35; 0.95]**	**0.55 [0.37; 0.83]**
D vs. A						0.69 [0.42; 1.15]	0.70 [0.44; 1.12]	**0.62 [0.41; 0.91]**
M vs. A						0.72 [0.46; 1.25]	0.73 [0.46; 1.18]	**0.66 [0.43; 0.997]**
R vs. A						**0.49 [0.30; 0.81]**	**0.50 [0.31; 0.81]**	**0.45 [0.30; 0.68]**
T vs. A						**0.43 [0.22; 0.82]**	**0.45 [0.25; 0.83]**	**0.44 [0.25; 0.76]**
V vs. A	2	2	0.80 [0.54; 1.17]	0.79 [0.00; 225.88]	0.79 [0.54; 1.17]	0.78 [0.49; 1.28]	0.80 [0.51; 1.24]	0.78 [0.54; 1.16]
SSRI vs. A						0.64 [0.40; 1.06]	0.65 [0.41; 1.03]	**0.60 [0.41; 0.91]**
TZA vs. A						0.83 [0.49; 1.37]	0.83 [0.50; 1.38]	0.94 [0.61; 1.47]
D vs. B						1.23 [0.91; 1.61]	1.22 [0.93; 1.60]	1.12 [0.91; 1.39]
M vs. B						1.30 [0.98; 1.69]	1.28 [0.93; 1.60]	1.19 [0.93; 1.52]
R vs. B						0.88 [0.65; 1.17]	0.87 [0.66; 1.16]	0.82 [0.64; 1.02]
T vs. B						0.77 [0.47; 1.28]	0.79 [0.50; 1.26]	0.81 [0.53; 1.18]
V vs. B	2		**1.43 [1.06; 1.91]**	1.43 [0.01; 289.17]		**1.39 [1.08; 1.79]**	**1.39 [1.09; 1.77]**	**1.43 [1.16; 1.69]**
SSRI vs. B						1.14 [0.88; 1.50]	1.14 [0.89; 1.46]	1.09 [0.90; 1.31]
TZA vs. B						**1.48 [1.04; 2.06]**	**1.45 [1.02; 2.04]**	**1.71 [1.27; 2.29]**

Table 6 continued

Comparison	N	N_I	$\texttt{DE}_{\texttt{Frequ}}$ OR [95 % − CI]	$\texttt{DE}_{\texttt{Bayes}}$ OR [95 % − CrI]	$\texttt{MTC}_{\texttt{Incon}}$ OR [95 % − CrI]	$\texttt{MTC}_{\texttt{Con}}$ OR [95 % − CrI]	`netmeta` OR [95 % − CI]	$\texttt{MTC}_{\texttt{SR}}$ OR [95 % − CrI]
M vs. D						1.05 [0.86; 1.29]	1.05 [0.85; 1.29]	1.07 [0.89; 1.26]
R vs. D						**0.71 [0.57; 0.91]**	**0.72 [0.58; 0.89]**	**0.73 [0.62; 0.92]**
T vs. D						**0.63 [0.39; 0.999]**	**0.65 [0.43; 0.99]**	0.72 [0.49; 1.04]
V vs. D	2	2	1.33 [0.93; 1.91]	1.35 [0.00; 428.80]	1.32 [0.95; 1.84]	1.14 [0.98; 1.36]	1.14 [0.97; 1.33]	**1.27 [1.10; 1.47]**
SSRI vs. D	9	2	1.02 [0.84; 1.24]	1.01 [0.78; 1.30]	1.30 [0.95; 1.80]	0.93 [0.81; 1.08]	0.93 [0.81; 1.08]	0.98 [0.86; 1.11]
TZA vs. D						1.20 [0.91; 1.57]	1.18 [0.88; 1.59]	**1.53 [1.16; 1.99]**
R vs. M						**0.68 [0.54; 0.86]**	**0.68 [0.55; 0.86]**	**0.68 [0.57; 0.82]**
T vs. M	1		0.63 [0.27; 1.48]	0.63 [0.26; 1.48]		**0.60 [0.38; 0.93]**	**0.62 [0.41; 0.95]**	**0.67 [0.46; 0.95]**
V vs. M	1	1	0.77 [0.47; 1.28]	0.77 [0.47; 1.28]	0.77 [0.45; 1.34]	1.08 [0.90; 1.28]	1.09 [0.91; 1.30]	1.19 [0.99; 1.47]
SSRI vs. M	11	10	0.93 [0.75; 1.14]	0.93 [0.74; 1.20]	0.92 [0.75; 1.13]	0.88 [0.75; 1.03]	0.89 [0.76; 1.05]	0.91 [0.78; 1.07]
TZA vs. M	1	1	1.44 [0.53; 3.91]	1.46 [0.53; 4.08]	1.51 [0.53; 4.35]	1.14 [0.85; 1.51]	1.13 [0.84; 1.52]	**1.43 [1.08; 1.87]**
T vs. R						0.88 [0.55; 1.41]	0.91 [0.59; 1.40]	0.99 [0.67; 1.39]
V vs. R						**1.59 [1.30; 1.92]**	**1.59 [1.31; 1.92]**	**1.72 [1.47; 2.08]**
SSRI vs. R	8	4	**1.26 [1.06; 1.49]**	1.26 [0.999; 1.56]	1.23 [0.88; 1.73]	**1.30 [1.09; 1.53]**	**1.30 [1.10; 1.55]**	**1.34 [1.16; 1.54]**
TZA vs. R						**1.68 [1.26; 2.25]**	**1.65 [1.21; 2.25]**	**2.09 [1.57; 2.75]**
V vs. T	2	1	1.40 [0.75; 2.62]	1.44 [0.00; 915.07]	**2.53 [1.22; 5.21]**	**1.82 [1.15; 2.86]**	**1.75 [1.17; 2.62]**	**1.75 [1.25; 2.63]**
SSRI vs. T						1.49 [0.94; 2.38]	1.43 [0.95; 2.16]	1.35 [0.97; 2.00]
TZA vs. T	1		**3.70 [1.63; 8.41]**	3.82 [1.70; 8.93]		**1.92 [1.19; 3.13]**	**1.82 [1.16; 2.87]**	**2.13 [1.47; 3.23]**

Table 6 continued

Comparison	N	N_I	$\mathtt{DE}_{\mathtt{Frequ}}$ OR [95 % − CI]	$\mathtt{DE}_{\mathtt{Bayes}}$ OR [95 % − CrI]	$\mathtt{MTC}_{\mathtt{Incon}}$ OR [95 % − CrI]	$\mathtt{MTC}_{\mathtt{Con}}$ OR [95 % − CrI]	netmeta OR [95 % − CI]	$\mathtt{MTC}_{\mathtt{SR}}$ OR [95 % − CrI]
SSRI vs. V	25	18	**0.83 [0.74; 0.94]**	**0.83 [0.72; 0.94]**	**0.85 [0.73; 0.98]**	**0.82 [0.73;0.91]**	**0.82 [0.73; 0.91]**	**0.77 [0.70; 0.85]**
TZA vs. V	10	9	1.02 [0.74; 1.40]	1.01 [0.68; 1.51]	1.15 [0.86; 1.52]	1.06 [0.84; 1.34]	1.04 [0.81; 1.34]	1.21 [0.96; 1.53]
TZA vs. SSRI						**1.29 [1.004; 1.64]**	1.27 [0.97; 1.66]	**1.56 [1.21; 2.01]**

95 %-CI/CrI not including the null effect are printed in **bold**.
A: Agomelatine; B: Bupropion; CrI: Credible interval; D: Duloxetine; CI: Confidence interval; M: Mirtazapine; N: Number of pairwise comparisons from studies; N_I: Number of pairwise comparisons from studies, which are included into $\mathtt{MTC}_{\mathtt{Incon}}$; OR: Odds ratio; P: Placebo; R: Reboxetine; SSRI: Selective serotonin reuptake inhibitor; T: Trazodone; TCA: Tri- and tetracyclic antidepressants; V: Venlafaxine

2 Code

In the following the `OpenBUGS` Code for the Bayesian models including the specification of the non informative prior distributions is presented. Additionally the starting values for the 3 Markov chains are given.

The Bayesian meta-analysis estimator

```
####### Model #######
model {
 for( i in 1 : Num ) {       # Num number of studies
  rc[i] ~ dbin(pc[i], nc[i]) # binominal likelihood
  rt[i] ~ dbin(pt[i], nt[i])
  logit(pc[i]) <- mu[i]      # Logit-link
  logit(pt[i]) <- mu[i] + delta[i]
  mu[i] ~ dnorm(0.0,1.0E-5) # prior baseline probability
  delta[i] ~ dnorm(d, tau)  # random effects
  }
 d ~ dnorm(0.0,1.0E-6)    # prior logOR
 tau <- 1/(sigma*sigma)  # prior variance between study results
 sigma ~ dunif(0,10)
}

####### Starting values for 3 Markov chains #######
inits1 <- list(d=0, sigma=1, mu=rep(0, 5), delta=rep(0, 5))
inits2 <- list(d=1, sigma=0.1, mu=rep(2, 5), delta=rep(1, 5))
inits3 <- list(d=1, sigma=2, mu=rep(-2, 5), delta=rep(-1, 5))
```

The MTC consistency model

```
####### Model #######
model {
 for(i in 1:ns) {        # ns Number of studies
  w[i,1] <- 0     # Adjustemnt for multiarm studies
                  # is 0 for the reference arm
  delta[i,1] <- 0 # Effect is 0 for the reference arm
                  # prior baseline probability
```

```
mu[i] ~ dnorm(0,.0001)
for (k in 1:na[i]) { # na Number of arms per study
 r[i,k] ~ dbin(p[i,k],n[i,k])  # Binomial likelihood
 logit(p[i,k]) <- mu[i] + delta[i,k] # Logit-link
       # Expected number of events
 rhat[i,k] <- p[i,k] * n[i,k]
       # Contribution to deviance
 dev[i,k] <- 2 * (r[i,k] * (log(r[i,k])-log(rhat[i,k]))
  + (n[i,k]-r[i,k]) * (log(n[i,k]-r[i,k])
  - log(n[i,k]-rhat[i,k])))
 }
       # Residual deviance for study i
resdev[i] <- sum(dev[i,1:na[i]])
for (k in 2:na[i]) {
       # Study-specific distribution logOR
 delta[i,k] ~ dnorm(md[i,k],taud[i,k])
       # Mean of logOR distribution
 md[i,k] <- d[t[i,k]] - d[t[i,1]] + sw[i,k]
       # Precision of logOR distribution
 taud[i,k] <- tau *2*(k-1)/k
       # Adjustment for multiarm studies
 w[i,k] <- (delta[i,k] - d[t[i,k]] + d[t[i,1]])
       # Cumulative adjustment for multiarm studies
 sw[i,k] <- sum(w[i,1:k-1])/(k-1)
 }
}
totresdev <- sum(resdev[])  # Residual deviance
    # Effect is 0 for reference intervention
d[1]<- 0
for (k in 2:nt)  {
    # Prior effects
 d[k] ~ dnorm(0,.0001)}
    # Prior variance between study results
 sd ~ dunif(0,10)
 tau <- pow(sd,-2)
# Pairwise OR and logORs for all pairwise comparisons
 for (c in 1:(nt-1)) {  for (k in (c+1):nt) {
```

```
    or[c,k] <- exp(d[k] - d[c])
    lor[c,k] <- (d[k]-d[c])
   }}
}

####### Starting values for 3 Markov chains #######
inits1 <- list(d=c(NA, rep(0, 4)), sd=1, mu=rep(0, 45))
inits2 <- list(d=c(NA, rep(1, 4)), sd=0.1, mu=rep(2, 45))
inits3 <- list(d=c(NA, rep(-1, 4)), sd=2, mu=rep(-2, 45))
```

The MTC inconsistency model

```
####### Model #######
model {
 for(i in 1:ns) {  # ns Number of studies
  delta[i,1]<-0    # Effect is 0 for the reference arm
                   # prior baseline probability
  mu[i] ~ dnorm(0,.0001)
  for (k in 1:na[i]) {   # na Number of arms per study
   r[i,k] ~ dbin(p[i,k],n[i,k])  # Binomial likelihood
   logit(p[i,k]) <- mu[i] + delta[i,k]  # Logit-link
                         # Expected number of events
   rhat[i,k] <- p[i,k] * n[i,k]
                         # Contribution to deviance
   dev[i,k] <- 2 * (r[i,k] * (log(r[i,k])-log(rhat[i,k]))
   + (n[i,k]-r[i,k]) * (log(n[i,k]-r[i,k])
   - log(n[i,k]-rhat[i,k])))
  }
                  # Residual deviance for study i
  resdev[i] <- sum(dev[i,1:na[i]])
  for (k in 2:na[i]) {
                   # Study-specific distribution logOR
   delta[i,k] ~ dnorm(d[t[i,1],t[i,k]] ,tau)
  }
 }
 totresdev <- sum(resdev[]) # Residual deviance
```

```
    # Prior for all mean effects
 for (c in 1:(nt-1)) {
  for (k in (c+1):nt) { d[c,k] ~ dnorm(0,.0001) }
 }
 sd ~ dunif(0,5)    # Prior SD between study results
 var <- pow(sd,2)  # Variance between study results
 tau <- 1/var
}

####### Starting values for 3 Markov chains #######
inits1 <- list(d=c(NA,NA,NA,NA,0,NA,NA,NA,0,0,NA,NA,
          0,0,0,NA,0,0,0,0), sd=1, mu=rep(0, 5))
inits2 <- list(d=c(NA,NA,NA,NA,2,NA,NA,NA,2,2,NA,NA,
          2,2,2,NA,2,2,2,2), sd=0.1, mu=rep(1, 5))
inits3 <- list(d=c(NA,NA,NA,NA,-2,NA,NA,NA,-2,-2,NA,NA,
          -2,-2,-2,NA,-2,-2,-2,-2), sd=2, mu=rep(-1, 5))
```
